# Betulinic Acid Attenuates T-2-Toxin-Induced Testis Oxidative Damage Through Regulation of the JAK2/STAT3 Signaling Pathway in Mice

**DOI:** 10.3390/biom9120787

**Published:** 2019-11-26

**Authors:** Jing Wu, Chenglin Yang, Juan Liu, Jiaxin Chen, Chao Huang, Ji Wang, Zengenni Liang, Lixin Wen, Jin-e Yi, Zhihang Yuan

**Affiliations:** 1 College of Veterinary Medicine, Hunan Agricultural University, Changsha 410128, Chinawjyjs@stu.hunau.edu.cn (C.Y.); jessienya@stu.hunau.edu.cn (J.L.); chenjiaxin92@126.com (J.C.); A390367297@stu.hunau.edu.cn (C.H.); sfwlx8015@sina.com (L.W.); 2 Department of Hunan Agricultural Product Processing Institute, Changsha 410128, China; cavepeoper@gmail.com

**Keywords:** T-2 toxin, betulinic acid, testis, oxidative stress, apoptosis, JAK2/STAT3 signaling pathway

## Abstract

T-2 toxin is one of the most toxic type A trichothecene mycotoxins in nature, and it exhibits reproductive toxicity. Betulinic acid (BA) is a natural pentacyclic triterpene compound found in species of Betula, and it has been reported to have antioxidant activity. The aim of the present study was to investigate the protective effect of BA on T-2-toxin-induced testicular injury in mice and explore its molecular mechanism. Sixty adult male mice were randomly divided into groups. The mice were pretreated orally with BA (0.25, 0.5, and 1.0 mg/kg) daily for 14 days, and the T-2 toxin (4 mg/kg body weight) was administered via intraperitoneal injection to induce oxidative stress after the last administration of BA. BA pretreatment significantly increased the secreted levels of testosterone and sperm motility. Moreover, BA pretreatment significantly increased the total antioxidant capacity (T-AOC), the activity of SOD and CAT, and the content of GSH, and it reduced the content of MDA. Furthermore, BA relieved testicular injury and reduced the number of apoptotic cells, and it significantly decreased the protein expression of Janus kinase 2 (JAK2), signal transducers and activators of transcription 3 (STAT3), caspsae-3, and Bcl-2-associated X protein (Bax). BA also increased the expression of B-cell lymphoma-2 (Bcl-2). We suggest that BA reduced the oxidative damage induced by T-2 toxin, and that these protective effects may be partially mediated by the JAK2/STAT3 signaling pathway.

## 1. Introduction

T-2 toxin is produced by *Fusarium piriformis*, and it is one of the most toxic A trichothecenes. Myotoxins are widely distributed in grains and agricultural products and have many toxic effects on humans and animals [1]. In China, the detection rate of T-2 toxin in cereals is as high as 80%, and the content can be as much as 735 ng/g [2]. T-2 toxin can cause toxicity of cells in the digestive system, immune system, and reproductive system, and it has teratogenic, carcinogenic, and mutagenic effects [3]. T-2 toxin can be absorbed through skin contact, intestinal absorption, respiration, and other channels. Accidental consumption of T-2-toxin-contaminated grain or feed can cause chronic and acute poisoning, which manifests as loss of appetite, vomiting, diarrhea, bloody stool, and so on [4]. It has been reported that T-2 toxin is associated with alimentary toxic aleukia [5]. It is the factor that causes Kashin–Beck disease and Keshan disease [6,7]. A variety of toxic effects induced by T-2 toxin have been confirmed, and reproductive system toxicity is a significant toxic effect [8]. Previous research in our lab has shown the apoptosis of rat ovarian granulosa cells following T-2 toxin exposure; the apoptosis occurs through the mitochondrial pathway, and is mediated by reactive oxygen species [9,10]; T-2 toxin also inhibits steroidogenic pathways in rat ovarian granulosa cells [11] and induces oxidative damage, DNA damage, and apoptosis in TM3 Leydig cells [12]. Additionally, oxidative stress has been confirmed in T-2-toxin-induced apoptosis. T-2 toxin can induce oxidative stress in human cervical cancer cells and GH3 cells, and it can activate the caspase signaling pathway to induce apoptosis [13]. Studies have shown that oxidative stress can reduce sperm quality and affect male fertility [14]. Using SerW3 cells as an in vitro model, T-2 toxin was shown to be able to destroy the blood–testis barrier (BTB) established by SerW3 cells [15]. Therefore, it is necessary to study the effects and mechanism of T-2 toxin regarding testicular injury and to find ways to alleviate it.

Betulinic acid (BA, 3β-hydroxy-lup-20(29)-en-28-oic acid) is a natural pentacyclic lupane-type triterpenoid widely distributed in plants; it was first isolated from the bark of birch tree (*Betula pendula* Roth) [16]. Accumulating experimental evidence has revealed that BA has various pharmacological activities, such as anti-inflammatory, antiviral, anticancer, parasiticidal, and anti-infectious effects [17]. As a biological molecule, BA exhibits both direct intrinsic and indirect antioxidant abilities by enhancing the antioxidant system in vitro and in vivo [18,19,20]. As reported in a previous study, BA is able to mitigate Dex-induced oxidative stress and apoptosis of splenocytes in mice [21]. Existing research reported that 1 mg/kg BA has a protective effect on dexamethasone-induced thymocyte apoptosis in a mouse model; the effect is due to BA reducing oxidative stress [22]. Similarly, BA pretreatment could also prevent alcohol-induced liver damage by increasing the activities of superoxide dismutase (SOD), catalase (CAT), and glutathione peroxidase (GSH-Px), and by reducing hepatic malondialdehyde (MDA) contents while increasing glutathione (GSH) levels, all of which occur in a dose-dependent manner [23]. However, little is known about the effect of BA on mycotoxin-induced damage to the reproductive system in vitro. In this study, we aimed to investigate the potential protective effect of BA on T-2-toxin-induced testis damage in mice and to demonstrate its molecular mechanisms.

## 2. Materials and Methods

### 2.1. Reagents and Chemicals

T-2 toxin was purchased from Puruibang Biological Engineering Co., Ltd. (Qingdao, Shandong, China). BA was purchased from Sigma-Aldrich (St. Louis, MO, USA).VE (Vitamin E) was bought from Sigma-Aldrich (St Louis, MO, USA). Trizol was purchased from Life Technologies (Ambion, Life Technologies Inc., Carlsbad, CA, USA), while the primescript RT reagent kit and SYBR Green I fluorescent dyes were purchased from Takara (Shiga, Japan). A BCA protein assay kit and assay kits for measuring total antioxidant capacity (T-AOC), superoxide dismutase (SOD), glutathione peroxidase (GSH), catalase (CAT), and malondialdehyde (MDA) were purchased from Nanjing Jiancheng Biotech (Nanjing, Jiangsu, China). Enhanced chemiluminescence (ECL) reagent was purchased from Nanjing KeyGen Biotech. Co., Ltd. (Nanjing, Jiangsu, China). A mouse testosterone ELISA kit was purchased from Wuhan Huamei Biological Engineering Co., Ltd. (Wuhan, Hubei, China). The primary antibodies for β-actin, STAT3, JAK2, Bax, Bcl-2, p-STAT3, p-JAK2, and caspsae-3 were obtained from Cell Signaling Technology (Boston, MA, USA).

### 2.2. Animals and Experimental Designs

A total of 60 20 ± 2 g, healthy, male Kunming mice were purchased from Hunan Silaikejingda Laboratory Animal Co., Ltd. (Changsha, Hunan, China). The doses of T-2 toxin and BA were selected based on our preliminary experiments and previous studies. The mice were randomly divided into six groups (*n* = 10/group): the negative control group; the T-2 toxin group (4 mg/kg); the low (0.25 mg/kg), medium (0.5 mg/kg), and high (1.0 mg/kg) dose of BA with T-2 toxin groups; and the positive control (VE at the dose of 100 mg/kg) with T-2 toxin group. BA was mixed in 1% soluble starch jelly at different doses and administered orally for 14 days. The control and the T-2 toxin groups were given 1% soluble starch jelly via the same route of administration, and the positive negative control group was given 100 mg/kg·BW of VE. After 14 days, the negative control group was intraperitoneally injected with a mixture of alcohol and PBS, and the other groups were intraperitoneally injected with T-2 toxin, which was dissolved in absolute ethanol and diluted with 0.9% normal saline. After fasting overnight for 15 h, all mice were euthanized by administration of chloral hydrate, and the testicular and epididymal tissue samples were quickly removed and collected. Testicular tissues were divided into four parts; one part was fixed in 10% formalin solution for histological analysis, and the remaining parts were frozen at −80 °C until further analysis. The experimental procedures complied with the Animal Care and Use Guidelines of China and were approved by the Animal Care Committee of Hunan Agricultural University and Use Committee at HUNAU (No. 43321811). No animals were subjected to unnecessary suffering in the present study.

### 2.3. Testosterone Enzyme-Linked Immunosorbent Assay

The testosterone levels in mice were measured using mouse testosterone enzyme-linked immunosorbent assay (ELISA) kits according to the manufacturer’s instructions.

### 2.4. Analysis of Sperm Motility and Morphology

The mouse epididymides were placed in a 24 well plate containing preheated PBS. When the sperm had fully left the epididymides, 10 µL of the sperm suspension was removed for detection on the sperm count plate. The number of surviving sperm and the total number of sperm were counted, and the sperm survival ratio was calculated (the number of surviving sperm/total number of sperm × 100%). Meanwhile, the sperm malformation rate ratio was calculated (the number of abnormal sperm/total number of sperm × 100%). 

### 2.5. Histopathology of Testicular Tissue 

Testicular tissues were fixed in 10% formalin solution for 2–3 days, successively dehydrated in different concentrations of alcohol, embedded in paraffin, sectioned, and cut into 5 µm thick slices. Slices were stained with hematoxylin and eosin (H&E) for histological examination. Morphological changes to the liver were detected using a Motic BA410 microscope with a magnification of 200× (Motic Incorporation, Xiamen, China), and images were examined by Image Pro-Plus Motic Med 6.0 software (Motic Incorporation, China). 

### 2.6. Measurements of Antioxidative Capacity in Testes

The activity of T-AOC, CAT, and SOD, and the content of MDA and GSH were determined using commercially available assay kits according to the manufacturer’s instructions.

### 2.7. Apoptosis Measured by Terminal Deoxynucleotidyl-Transferase-Mediated dUTP Nick End Labeling (TUNEL) Staining 

A TUNEL assay was used to detect apoptosis in the testicular tissues of mice. The testicular tissues were cut into 4 µm thick slices and washed with PBS. They were then permeabilized with proteinase K for 30 min at 37 °C and were rinsed three times in PBS for 5 min at room temperature. The slices were incubated with TdT and dUTP at 37 °C for 2 h. After rinsing the tissue slices with PBS three times, they were analyzed via fluorescence microscopy (Motic, BA410, China). Images were calculated from five randomly selected fields of each slice with a magnification of 200×.

### 2.8. Western Blot Analysis

The testicular tissues were removed and placed into a centrifuge tube containing RIPA lysis buffer and phenylmethanesulfonyl fluoride (PMSF), and the homogenate was centrifuged at 12,000 rpm for 5 min at 4 °C. After collecting the supernatants for western blots, a BCA protein assay kit was used to determine the total protein concentrations. Sample proteins were separated via 10% sodium dodecyl sulfate polyacrylamide gel electrophoresis (SDS-PAGE) and transferred to PVDF membranes (Millipore, Bedford, MA, USA). After blocking with a 5% nonfat milk–TBST buffer for 1 h at 4 °C, the membranes were incubated with the following primary antibodies overnight at 4 °C: Bax, Bcl-2, caspsae-3, STAT3, p-STAT3, JAK2, and p-JAK2. After incubation, the membranes were washed three times with TBST at room temperature and incubated with the appropriate secondary antibody for 1 h. Finally, the protein bands were visualized using ECL reagent, and were quantitatively analyzed using Image-Pro Plus 6.0 software (BioRad, Hercules, CA, USA). 

### 2.9. Statistical Analysis

The experimental data of each group were analyzed by SPSS 21.0 statistical software (Release 21.0; SPSS, Inc., Chicago, IL, USA), using one-way ANOVA with the LSD post-hoc analysis. The results are expressed as the mean ± SEM. *p* values < 0.05 were considered statistically significant, while *p* values < 0.01 were considered highly significant. 

## 3. Results

### 3.1. BA Repaired T-2-Toxin-Induced Testis Injury in Mice

**Figure 1:**
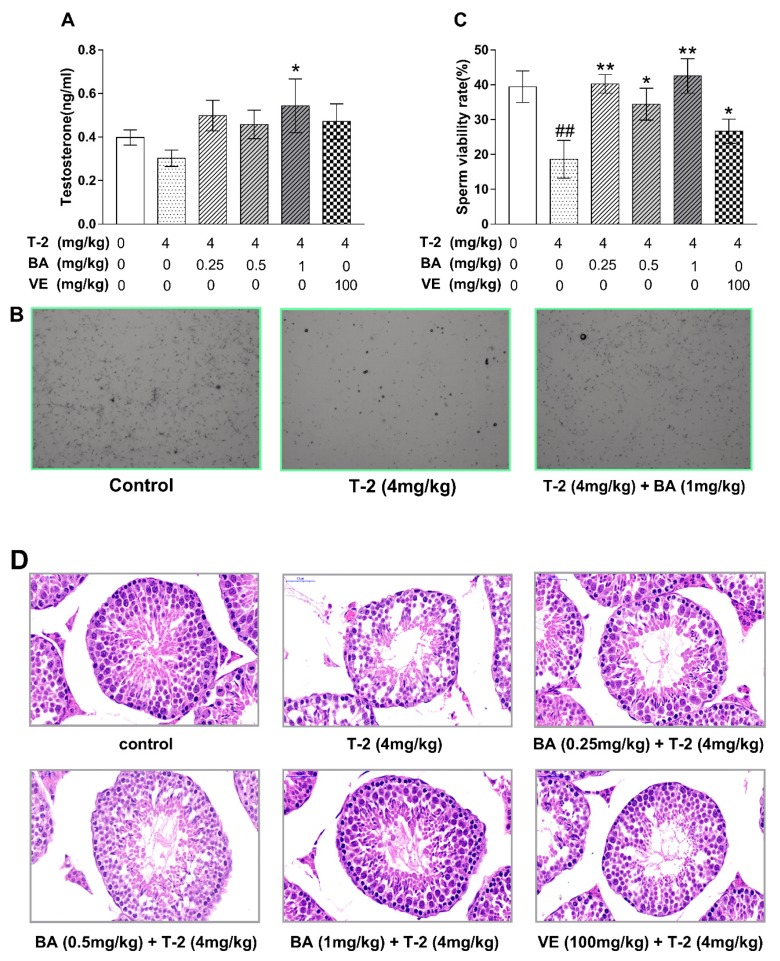
The effects of betulinic acid (BA) on testosterone secretion, quality of sperm and testis structural damage induced by T-2 in mice. (**A**) Testosterone secretion; (**B**) sperm morphology photo (4×); (**C**) sperm viability rate; (**D**) testis morphology of structural damage (40×). The values are presented as the mean ± SEM. Compared with the control group, ^##^ *p* < 0.01, and compared with T-2 toxin group, * *p* < 0.05, ** *p* < 0.01.

To determine what beneficial effects BA had on testis injury induced by T-2 toxin in mice, we assayed serum testosterone levels, sperm viability, sperm morphology, and histopathological changes in testis tissues (Figure 1). As shown in Figure 1A, treatment with T-2 toxin reduced the serum level of testosterone in testicular tissues compared to the negative control group. Moreover, pretreatment with BA at different doses increased serum testosterone levels compared to the T-2 toxin group. The percentage of viable sperm was significantly reduced in mice exposed to T-2 toxin compared to those in the negative control group (Figure 1B,C). However, pretreatment with low and high doses of BA significantly increased the percentage of viable sperm compared with the T-2 toxin group, and the medium dose of BA had a higher percentage of sperm survival than the T-2 toxin group; the difference in sperm survival for the VE group was obvious (Figure 1B). We measured the histopathological changes in the testis tissues, and the H&E staining results are shown in Figure 1D. Compared with the negative control group, the seminiferous tubule structures were seriously damaged and considerable cell necrosis was found after treatment with T-2 toxin. Compared with the T-2 toxin group, the structural damage of the seminiferous tubules and the areas of damaged cells were reduced in a dose-dependent manner following pretreatment with BA. These results indicate that BA could effectively alleviate testis injury induced by T-2 toxin.

### 3.2. The Effect of BA on Testicular Antioxidant Capacity Induced by T-2 Toxin in Mice 

**Figure 2:**
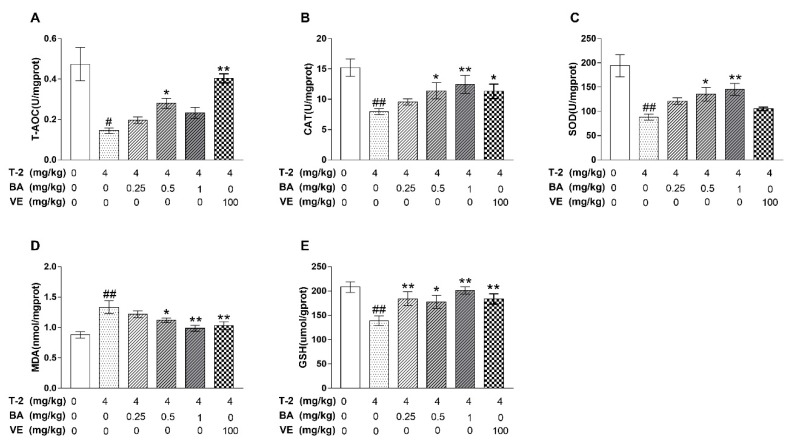
The effects of BA on oxidative stress induced by T-2 in mice. (**A**) Total antioxidant capacity (T-AOC) level; (**B**) catalase (CAT) level; (**C**) superoxide dismutase (SOD) level; (**D**) malondialdehyde (MDA) content; (**E**) glutathione peroxidase (GSH) content. The values are presented as the mean ± SEM. Compared with the control group, ^#^ *p* < 0.05, ^##^ *p* < 0.01, and compared with T-2 toxin group, * *p* < 0.05, ** *p* < 0.01.

The increase in antioxidant capacity is indicated by the improvement of antioxidant enzyme activity and GSH content. MDA is often used as an indicator of lipid peroxidation in tissues. As shown in Figure 2, the administration of T-2 toxin alone markedly decreased the levels of CAT, SOD, GSH, and T-AOC, but significantly increased the level of MDA compared to the negative control group. Compared with the T-2 toxin group, treatment with BA significantly enhanced the activities of CAT and SOD in a dose-dependent manner. Additionally, pretreatment with BA resulted in higher T-AOC activity than what was observed in the T-2 toxin group, and the highest T-AOC activity was observed in the group with the medium dose of BA. Compared with the T-2 toxin group, after receiving low and high doses of BA pretreatment, the level of GSH was significantly increased. However, pretreatment with BA decreased the level of MDA in a dose-dependent manner compared to the levels in the T-2 toxin group. In conclusion, these findings showed that BA could relieve T-2-induced oxidative damage in testis tissues.

### 3.3. The Effect of BA on the Apoptosis of Testicular Cells and Apoptosis-Related Protein Expression Induced by T-2 Toxin in Mice

**Figure 3:**
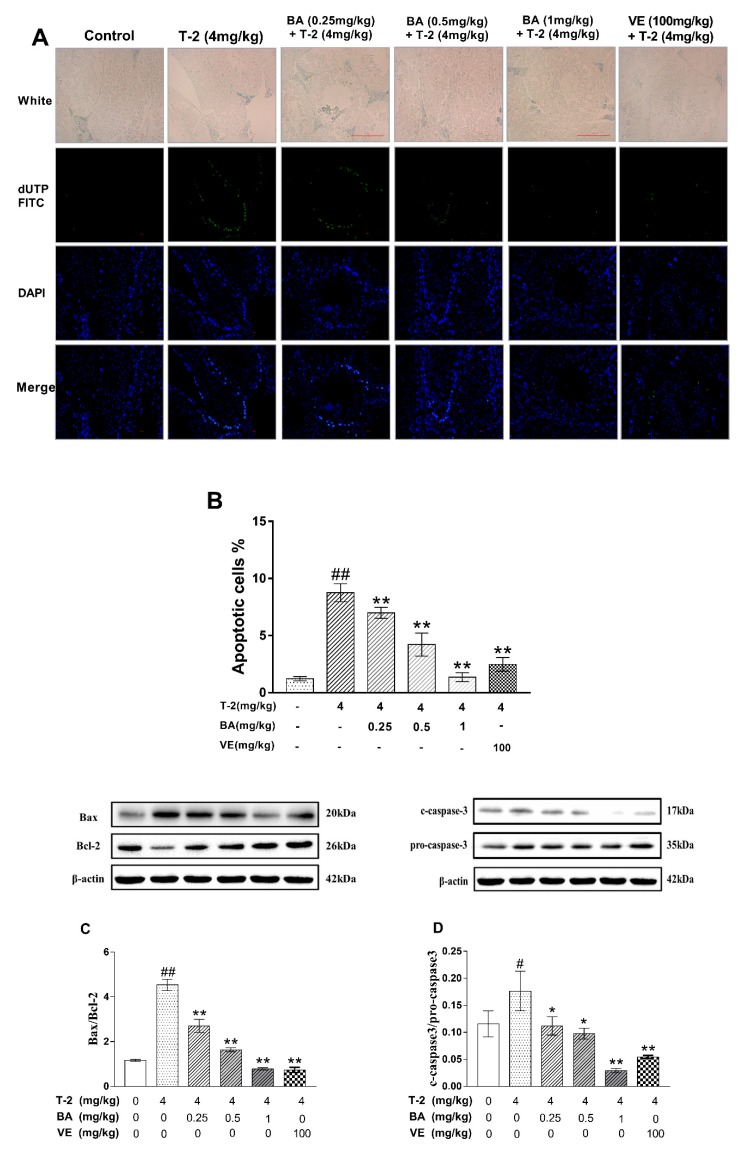
The effect of BA on testicular cell apoptosis induced by T-2 in mice and activity of apoptosis-related protein in Bcl-2, Bax, andcaspase-3. (**A**) Testicular cell apoptosis (TUNEL method); (**B**) apoptotic cel % (number of apoptotic cells/number of living cells × 100%). (**C**) Expression of Bcl-2 and Bax were analyzed by western blot; the ratio of Bax/Bcl-2 was significantly increased in mice by T-2 toxin treatment; (**D**) the expression of protein caspase-3 was analyzed by western blot and the ratio of cleaved-caspase-3/pro-caspase-3 was significantly increased by T-2 toxin treatment. The values are presented as the mean ± SEM. Compared with the control group, ^#^ *p* < 0.05, ^##^ *p* < 0.01, and compared with T-2 toxin group, * *p* < 0.05, ** *p* < 0.01.

Apoptosis in testicular tissue was detected by TUNEL staining. Non-apoptotic cells were stained with DAPI, and apoptotic cells were stained with dUTP-FITC. The results are shown in Figure 3A. Compared with the negative control group, the percentage of apoptotic cells was significantly increased in the T-2 toxin group (Figure 3B). However, pretreatment with BA significantly diminished the number of apoptotic cells in a dose-dependent manner compared with the levels from the T-2 toxin group. To determine the protective mechanism of BA on T-2-toxin-induced apoptosis, the expression levels of Bcl-2 family proteins and caspsae-3 were detected by western blot (Figure 3C,D). Compared with the negative control group, the ratio of the pro-apoptotic protein Bax to the anti-apoptotic protein Bcl-2 was markedly increased, and the levels of cleaved caspase-3 increased after administration of the T-2 toxin. Nevertheless, pretreatment with BA significantly decreased the Bax/Bcl-2 ratio and cleaved caspase-3/pro-caspase-3 in a dose-dependent manner compared with the T-2 toxin group. These results showed that BA could inhibit T-2-toxin-induced apoptosis through the mitochondrial signaling pathway.

### 3.4. The Effects of BA on the Expression of Apoptosis-Related Proteins in the JAK2/STAT3 Signaling Pathway Induced by T-2 Toxin in Mice

**Figure 4:**
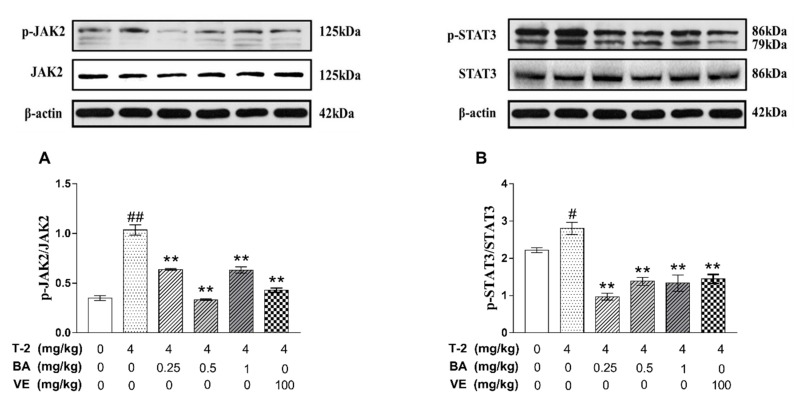
The effects of BA on activity of protein in JAK2, STAT3. (**A**) The phosphorylation of JAK2 was analyzed by western blot, and the ratio of p-JAK2/JAK2 was significantly increased by T-2 toxin treatment; (**B**) the phosphorylation of STAT3 was analyzed by western blot, and the ratio of p-STAT3/STAT3 was significantly increased by T-2 toxin treatment. The values are presented as the mean ± SEM. Compared with the control group, ^#^ *p* < 0.05, ^##^ *p* < 0.01, and compared with T-2 toxin group, ** *p* < 0.01.

We measured JAK2, p-JAK2, STAT3, and p-STAT3 protein expression in the testis tissues by western blot. After treatment with T-2 toxin, the JAK2 and STAT3 proteins were phosphorylated, and the ratios of p-JAK2/JAK2 and p-STAT3/STAT3 were higher than those in the negative control group. Compared with the T-2 group, BA pretreatment significantly reduced the ratios of p-JAK2/JAK2 and p-STAT3/STAT3. These results indicate that BA could alleviate apoptosis of testicular cells and testis injury induced by the T-2 toxin through the JAK2/STAT3 signaling pathway (Figure 4).

## 4. Discussion

The male reproductive system is required for fertility. Approximately 15% of couples around the world suffer from infertility, of which approximately 50% is caused by male reproductive dysfunction [24]. Testicular injury is an important cause of male reproductive problems. Testosterone levels are closely related to spermatogenesis and secondary sex characteristics [25], and testosterone is mainly synthesized and secreted by Leydig cells [26]. The testis is predominantly composed of spermatogenic cells, Sertoli cells, and Leydig cells. During spermatogenesis, spermatogenic cells produce sperm, and they are coregulated by Sertoli cells and Leydig cells [27]. Sperm is produced by and develops in the testicles, and when the testicles are damaged, it inevitably affects the male’s reproductive ability. Cadmium poisoning can aggravate testicular injury and affect the secretion of testosterone, possibly by changing the LHR, 17 alpha-hydroxylase, and eNOS expression levels of testicular stromal cells [28]. Sperm viability can objectively reflect the development of the testis. Testosterone plays an important role in spermatogenesis through development, maintaining the blood–testis barrier, regulating meiosis, supporting the adhesion of sperm and supporting cells, and regulating the release of mature sperm [29].

T-2 toxin is a common trichothecene mycotoxin found in the environment. It has reproductive toxicity and can cause testicular injury through oxidative stress [30]. Oxidative stress is an important factor affecting reproductive function. Oxidative stress can induce apoptosis by activating the mitochondrial signaling pathway. Studies have shown that oxidative stress can alter the amount of iron and calcium in sperm, reducing sperm quality [31]. As a marker of lipid peroxidation, MDA can reflect the degree of oxidative stress. Oxidative stress can also be assessed by detecting the testis content of MDA and GSH, as well as the activities of T-AOC, CAT, and SOD. In our study, we found that T-2 toxin significantly reduced the activity of T-AOC, CAT, and SOD, and reduced the content of GSH while it increased the content of MDA; these data are consistent with previous research results [32]. In line with this, the present study showed that T-2 toxin also caused testicular damage in mice, decreased the number of mesenchymal cells, and reduced serum testosterone levels and sperm motility, which was consistent with previous studies [32]. This suggests that T-2 toxin induced testis damage via oxidative stress in mice.

BA is a natural product that possesses serious biological and pharmacological properties. The pentacyclic triterpene nucleus of BA comprises six isoprene units that exhibit a variety of biological activities, including antioxidative activity [33,34]. In this study, after giving the mice oral doses of 0.25, 0.5, or 1 mg/kg BA, or 100 mg/kg VE before exposure to T-2 toxin, the activities of CAT and SOD in the testis were higher, and the content of MDA in the testis was lower than in mice without BA treatment. As a result, the mice with no BA treatment showed more severe disruption of testicular structure than those mice administered oral BA. These results indicate that BA protected mice from T-2-toxin-induced testicular structural damage by mitigating oxidative stress.

There is evidence suggesting that T-2 toxin could induce germ cell apoptosis by regulating the expression of Bcl-2 family and caspase family apoptosis-related genes in the mitochondrial signaling pathway. The expression of caspase-8 mRNA was increased, suggesting that the apoptosis of germ cells may be related to the death receptor pathway [32]. The Bcl-2 gene family regulates apoptosis through the mitochondrial pathway. Bax is an important pro-apoptotic gene in the Bcl-2 family. An increased ratio of Bax/Bcl-2 is a key indicator of the activation of the apoptotic process [35]. The caspase-3 family also plays an important role in mediating apoptosis, and caspase-3 is an executive factor for apoptosis [36]. JAK2/STAT3 is an important signaling pathway involving the Janus kinase (JAK)/signal transducer and activator of transcription (STAT) family, which is involved in cell proliferation, apoptosis, immunity, and inflammation [37]. For example, Zhang et al. reported that suppression of the JAK2/STAT3 pathway alleviated DDP-induced lung cancer in mice with improved oxidative stress and apoptosis [38]. Wu et al. demonstrated that suppression of the JAK2/STAT3 pathway alleviated the apoptosis of liver cancer cells [39]. In our experiments, we showed that T-2 toxin increased the phosphorylation ratio of JAK2 and STAT3 in mouse testes, which was consistent with previous studies [40]. Increases in the ratio of Bax/Bcl-2 and the expression of caspase-3 in the testis of mice with T-2 toxin exposure were also observed in this study. Consistently, the TUNEL assay confirmed that T-2 toxin induced apoptosis in testicular tissue. This suggests that activation of JAK2/ STAT3 signaling and the Bax/Bcl-2 pathway are involved in T-2-toxin-induced apoptosis.

The results of this work showed that BA supplementation reversed the ratios of p-JAK2/JAK2, p-STAT3/STAT3, and Bax/Bcl-2, and reduced the activation of caspase-3, which was confirmed by the TUNEL results; these data suggest that T-2-toxin-induced apoptosis was blocked by BA treatment. Accumulating evidence has demonstrated that BA is able to induce apoptosis in a broad variety of cell types, including human cervical cancer cells, breast cancer cells, and HT-29 colorectal cancer cells [41,42,43]. In our experiment, BA acted as an antiapoptotic agent in the testes of mice treated with T-2 toxin. These results prove that BA acts as a selective inhibitor of cancer, whereas it plays an antiapoptotic role in noncancerous tissues.

## 5. Conclusions

**Figure 5:**
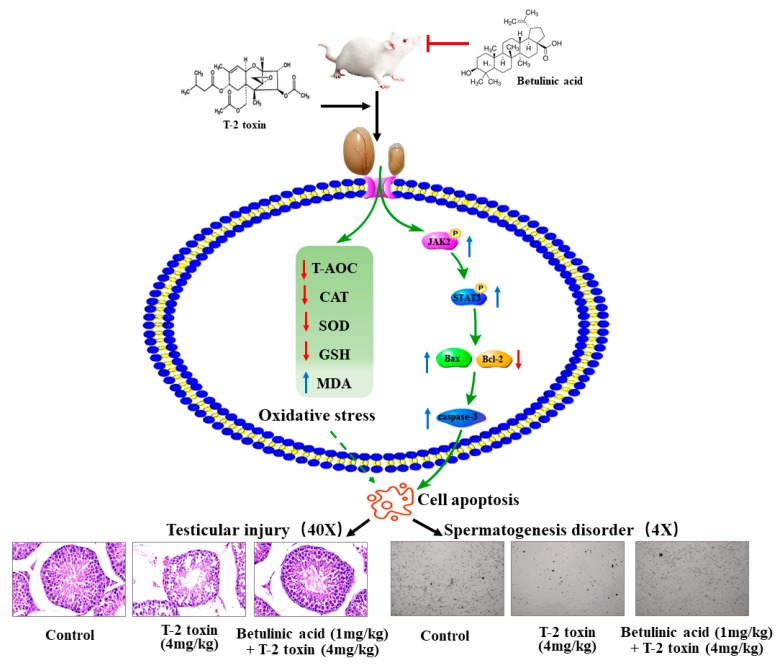
A schematic diagram of the proposed mechanisms by which betulinic acid ameliorates T-2-toxin-induced oxidative stress and diminishes T-2-toxin-induced apoptosis via the JAK2/STAT3 and BAX/BCL-2/caspase-3 pathways.

In summary, this work showed that T-2 toxin exposure induced testicular structural damage, decrease the serum testosterone content, and decrease sperm viability via triggering oxidative stress in the testis and promoting apoptosis in mouse testes. The natural product betulinic acid has the ability to ameliorate T-2-toxin-induced oxidative stress and diminish T-2-toxin-induced apoptosis via the JAK2/STAT3 and BAX/BCL-2/caspase-3 pathways (Figure 5). These outcomes protect the testis from damage and increase the serum testosterone content and sperm viability. This work provides insight into the potential of betulinic acid as an antimycotoxin agent that protects the reproductive system.
